# Supplementary material for: Food amyloid fibrils are safe nutrition ingredients based on in-vitro and in-vivo assessment
Source: Nat Commun. 2023 Oct 26;14:6806. doi: 10.1038/s41467-023-42486-x (PMC10603083; doi:10.1038/s41467-023-42486-x)
Supplement: Supplementary file 1 — supplementary information [file 41467_2023_42486_MOESM1_ESM.pdf]

Supplementary Information for

Food Amyloid Fibrils are Safe Nutrition Ingredients based on *In-vitro* and *In-vivo* Assessment

Dan Xu[1,2,†], Jiangtao Zhou[2,†,*], Wei Long Soon[2,3], Ines Kutzli[2], Adrian Molière[4], Sabine Diedrich[2], Milad Radiom[2,5], Stephan Handschin[6], Bing Li[1], Lin Li[1], Shana J. Sturla[2], Collin Y. Ewald[4], and Raffaele Mezzenga[2,7,*]

[1] School of Food Science and Engineering, Guangdong Province Key Laboratory for Green Processing of Natural Products and Product Safety, Engineering Research Center of Starch and Plant Protein Deep Processing, Ministry of Education, South China University of Technology, 381 Wushan Road, Tianhe District, Guangzhou, 510640, China

[2] Institute of Food, Nutrition and Health (IFNH), Department of Health Sciences and Technology (HEST), ETH Zurich, Zürich 8092, Switzerland

[3] Center for Sustainable Materials (SusMat), School of Materials Science and Engineering, Nanyang Technological University, 639798 Singapore

[4] Institute of Translational Medicine, Department of Health Sciences and Technology (HEST), ETH Zurich, Schwerzenbach, Switzerland

[5] Laboratory of Food Immunology, Institute of Food, Nutrition and Health, ETH Zürich, Zürich, Switzerland

[6] Scientific Center for Optical and Electron Microscopy (ScopeM), ETH Zurich, Otto-Stern-Weg 3, 8093 Zurich, Switzerland

[7] Department of Materials, ETH Zurich, Zürich 8092, Switzerland

† These authors contributed equally

*Corresponding authors: jiangtao.zhou@hest.ethz.ch, raffaele.mezzenga@hest.ethz.ch

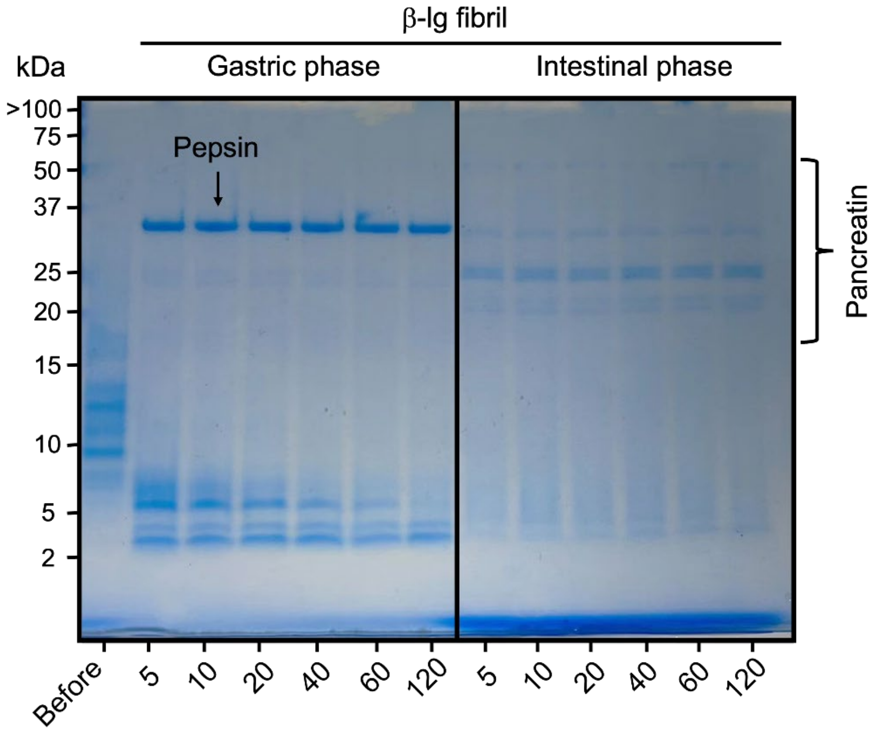

Supplementary Figure 1. A higher percentage SDS-PAGE gel (15%) of β-lg amyloid digestion showing a higher resolution at the lower molecular weight components during intestinal digestion, demonstrating full hydrolysis after complete gastrointestinal digestion.

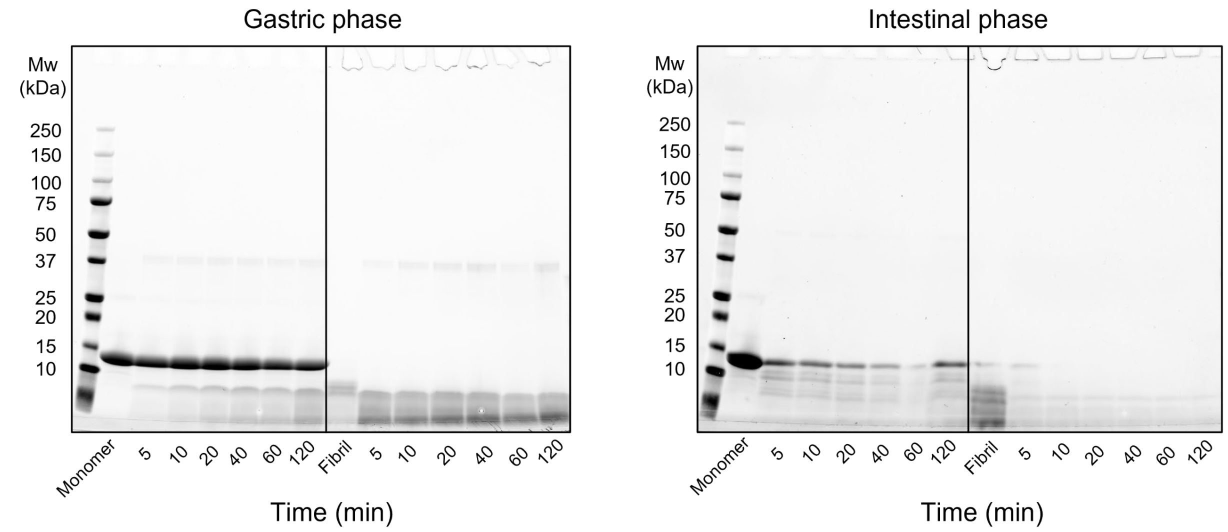

Supplementary Figure 2. Digestion fate of lysozyme in vitro. The SDS-PAGE of the gastric (left panel) and intestinal (right panel) digestion phases. In each panel, the lysozyme monomer is in the left part and amyloid fibril is in the right part.

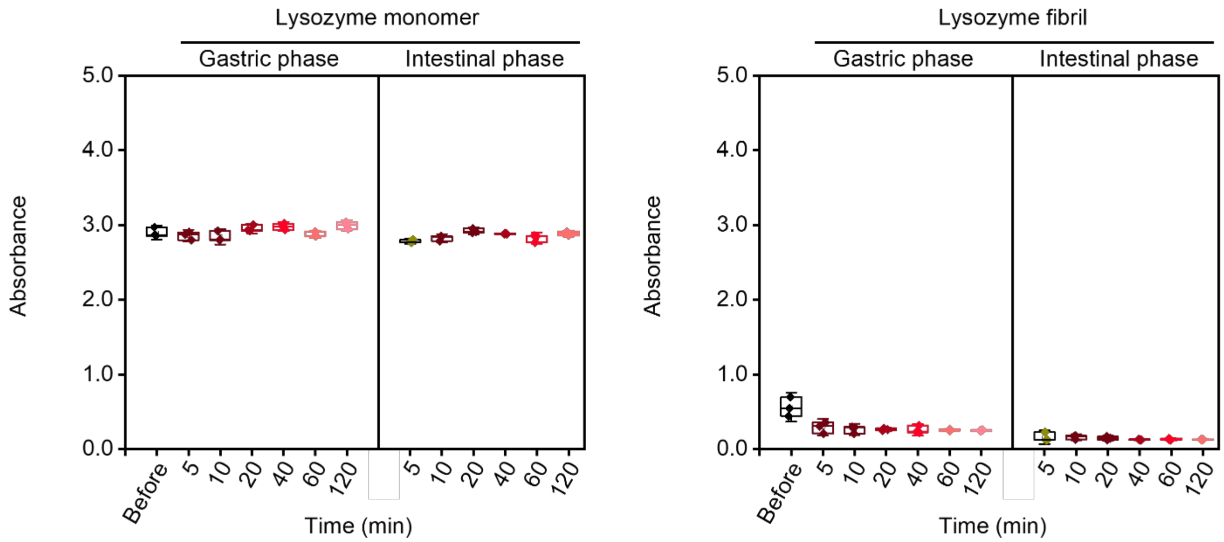

Supplementary Figure 3. ELISA antigenicity assay of lysozyme monomer and amyloid fibrils over the course of gastrointestinal digestion. The boxes cover the range of 25-75 percentage, the whisker lines and centre lines refer to SD and median value respectively. The plots are showed as mean values ± standard deviation. N=3 independent experiments.

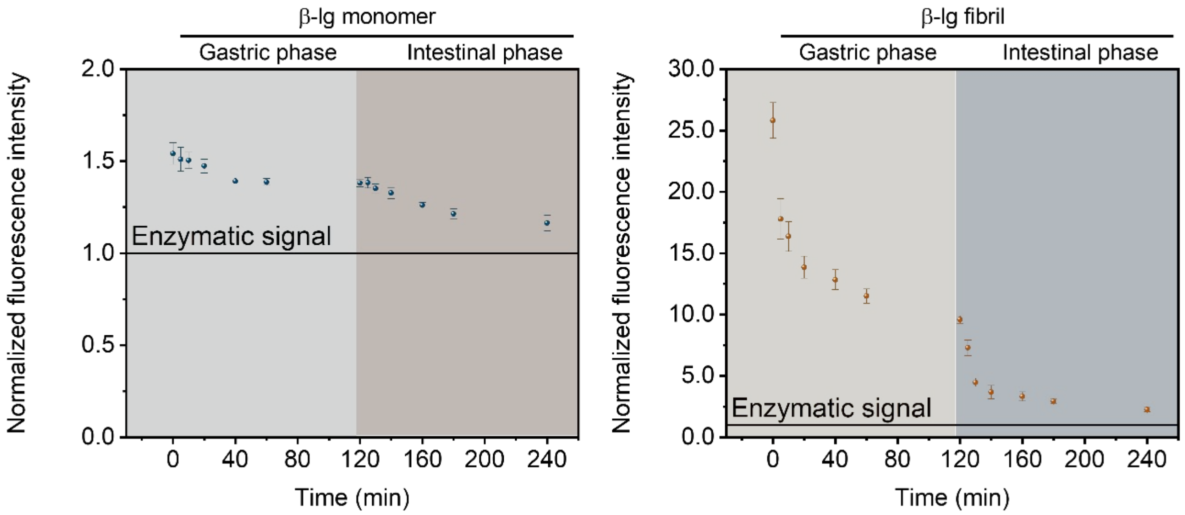

Supplementary Figure 4. ThT fluorescence assay of β-lg monomer and amyloid fibrils over the course of gastrointestinal digestion. Enzymatic signal refers to the background contribution intensity from digestive enzymes. N=3 independent experiments.

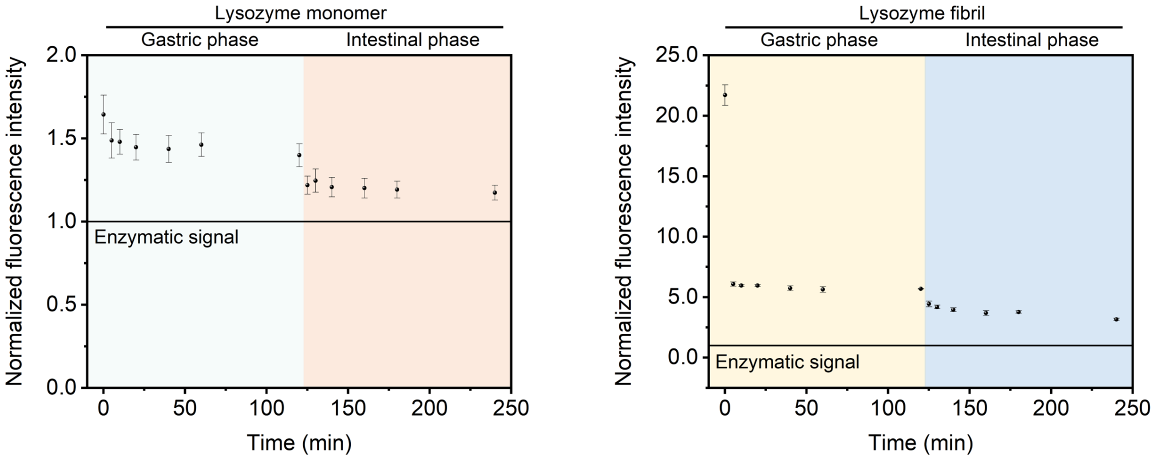

Supplementary Figure 5. TO fluorescence assay of lysozyme monomer and amyloid fibrils over the gastrointestinal digestion. Enzymatic signal refers to the background contribution intensity from digestive enzymes. Error bars represent SD. N=3 independent experiments.

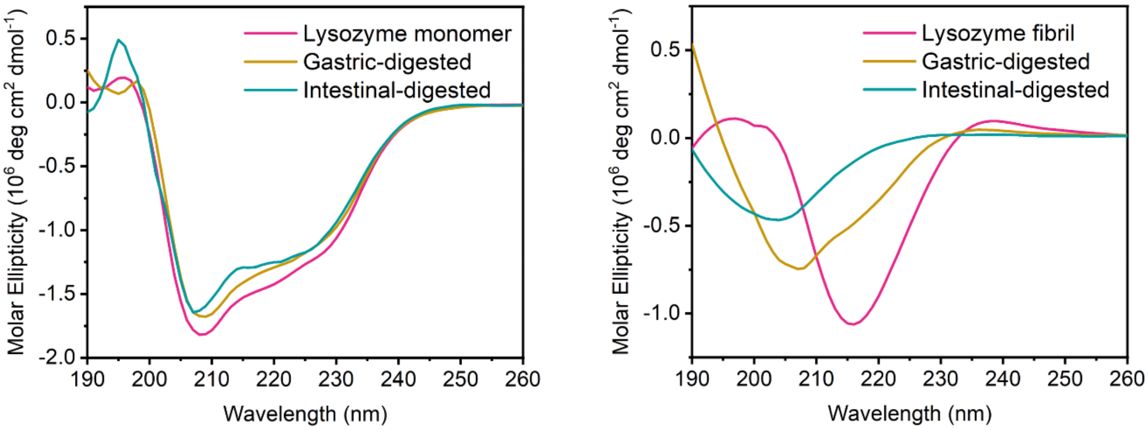

Supplementary Figure 6. Circular dichroism spectra of lysozyme monomer and amyloid fibrils after gastric and intestinal digestion. Lysozyme monomer is in the left panel, and lysozyme fibril is in the right panel.

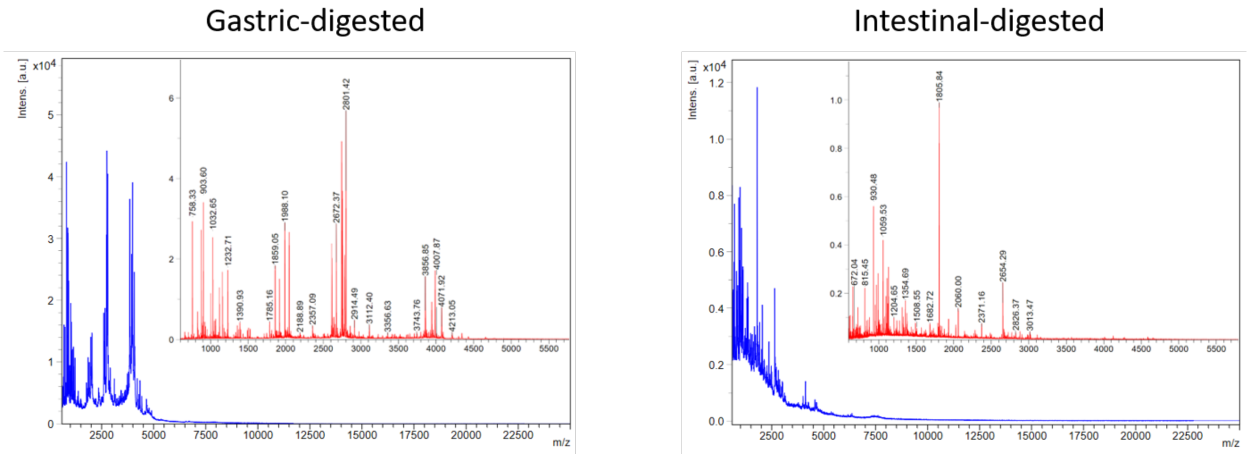

Supplementary Figure 7. MALDI-MS spectra of 10 kDa filter permeates gastric and intestinal digested β-lg amyloid fibrils. Left panel shows the permeate are gastric digestion, and the right panel after additional intestinal digestion. The insets are in reflector mode.

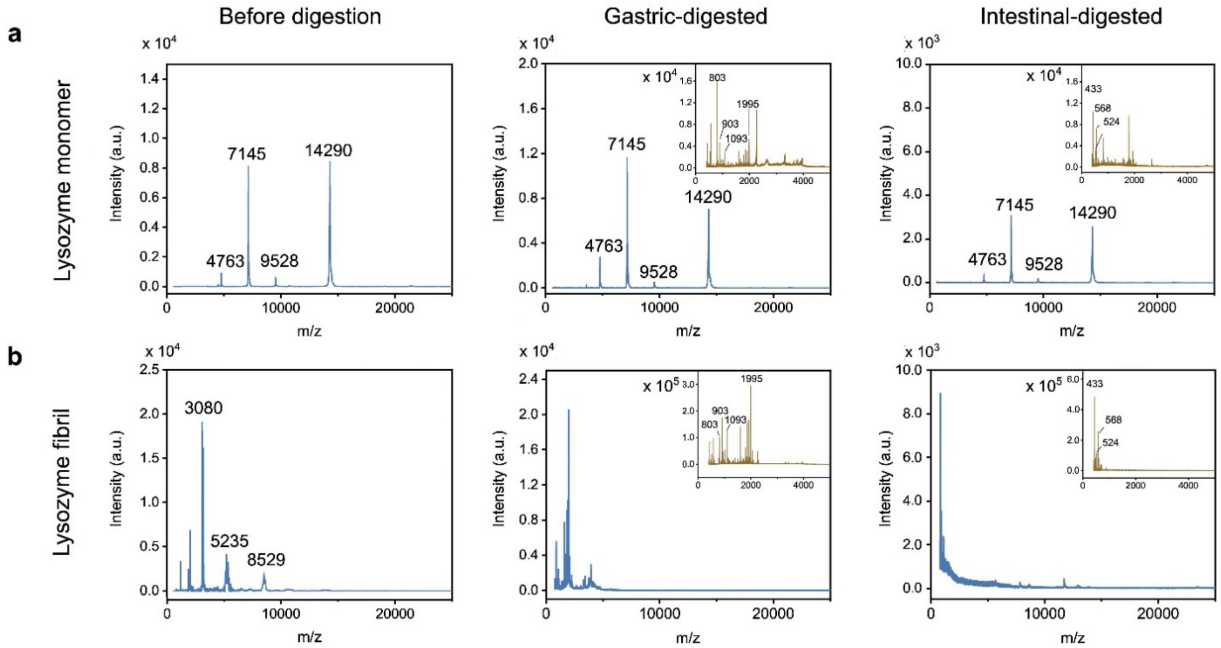

Supplementary Figure 8. MALDI-MS spectra of lysozyme monomer (a) and amyloid fibrils (b) digested products. The blue plots were obtained in linear mode in the range of 0.5 to 25 kDa, and the inserted plots were recorded in reflection mode in the range of 0.4 to 5 kDa.

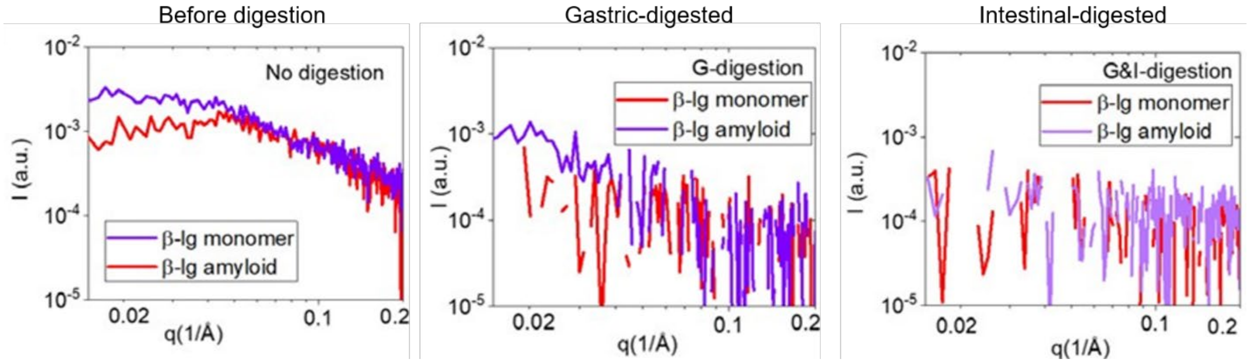

Supplementary Figure 9. Background subtracted SAXS profiles of β-lg monomer and amyloid fibril before digestion, after gastric digestion, and intestinal digestion.

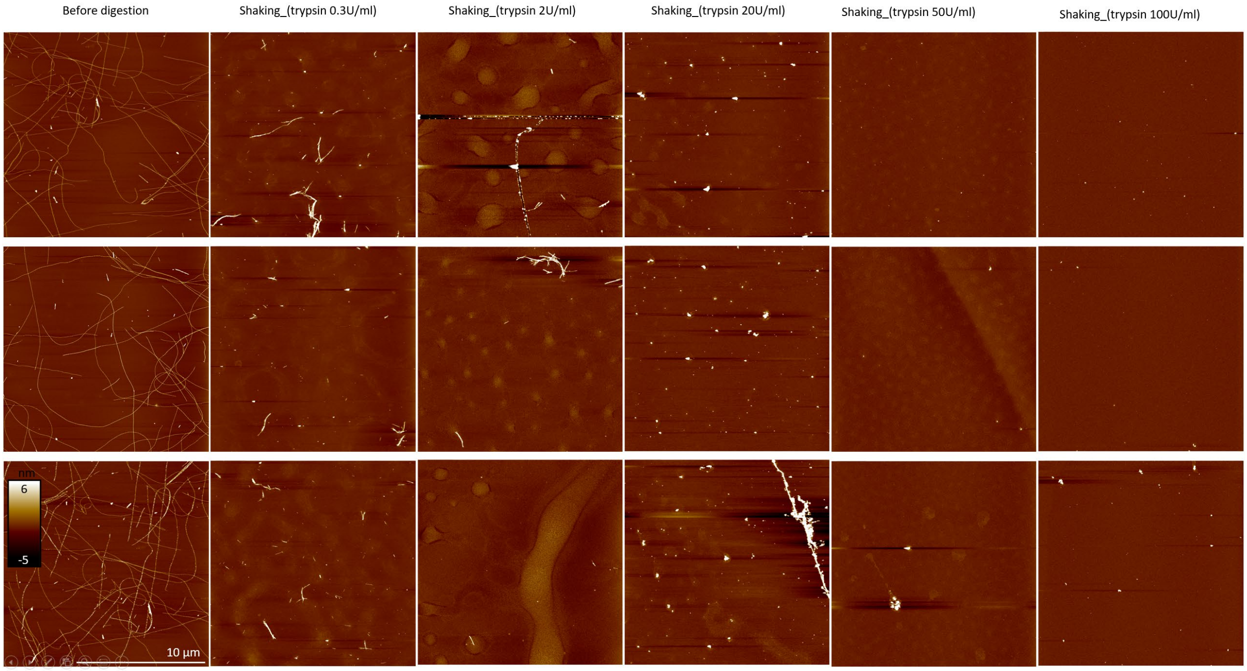

Supplementary Figure 10. AFM images of β-lg amyloid fibrils after gastrointestinal digestion in the INFOGEST protocol under the shaking condition, at different concentrations of trypsin ranging from 0.3 U/mL to 100 U/mL. The fibrils are fully digested with trypsin concentration of around 50 U/mL. AFM images were collected in random locations on the mica surface at a size of 15 by 15 µm. N=3 independent experiments.

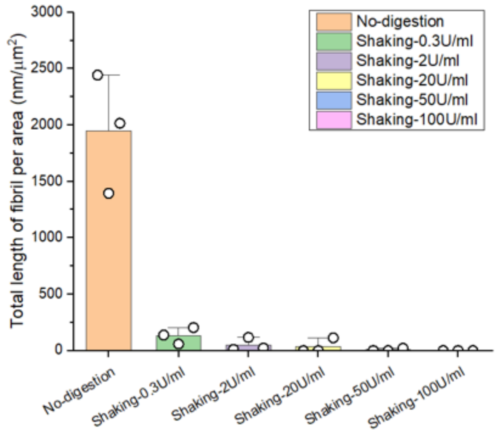

	Total length of fibril per area (nm/μm^2)			Average (nm/μm^2)
No digestion	2015.20	1395.10	2442.46	1950.92±526.63
Shaking-0.3U/ml	58.38448	137.65216	204.30097	133.45±400.34
Shaking-2U/ml	116.17881	10.13063	21.94973	49.42±58.12
Shaking-20U/ml	0	0	113.21422	37.73807
Shaking-50U/ml	0	0	19.72806	6.57602
Shaking-100U/ml	0	0	0	0

Supplementary Figure 11. AFM statistical analysis of β-lg amyloid fibril length after gastrointestinal digestion in the INFOGEST protocol under the shaking condition. N=3 independent experiments. The total length of fibril per area was calculated at different concentrations of trypsin ranging from 0.3 U/mL to 100 U/mL. Data are presented as mean±SD.

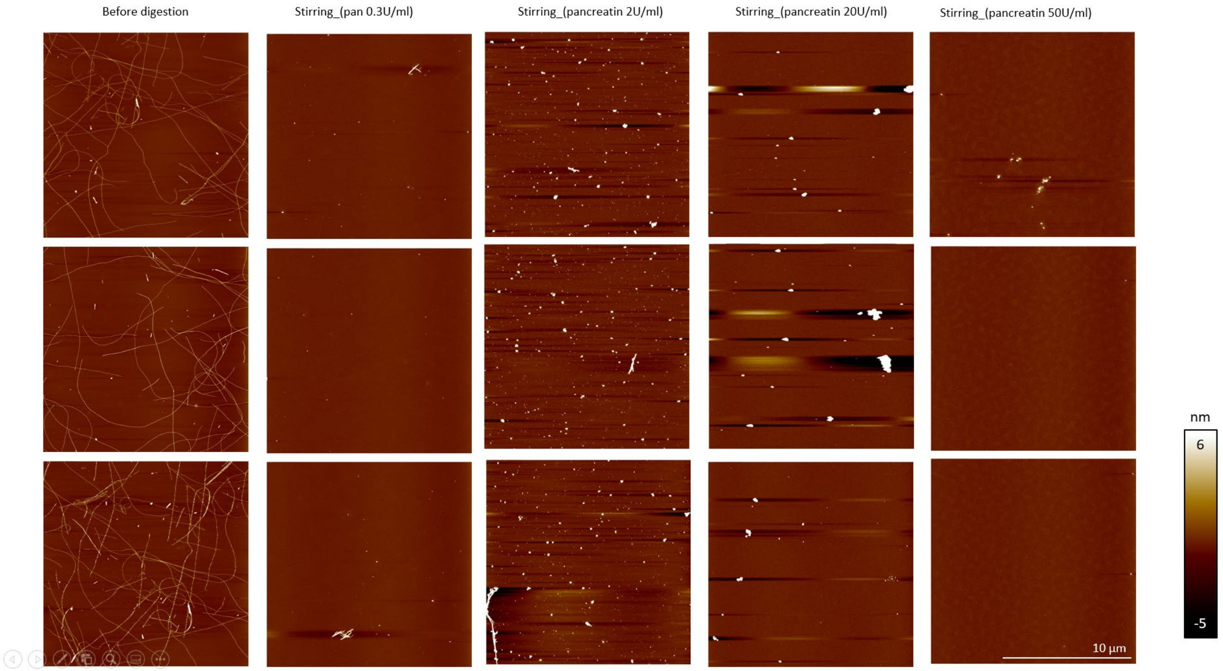

Supplementary Figure 12. AFM images of β-lg amyloid fibrils after gastrointestinal digestion in the INFOGEST protocol under the moderate stirring condition, at different concentrations of trypsin ranging from 0.3 U/mL to 100 U/mL. The fibrils are fully digested with the trypsin concentration of around 20 U/mL. AFM images were collected in random locations on the mica surface at a size of 15 by 15 μm. N=3 independent experiments.

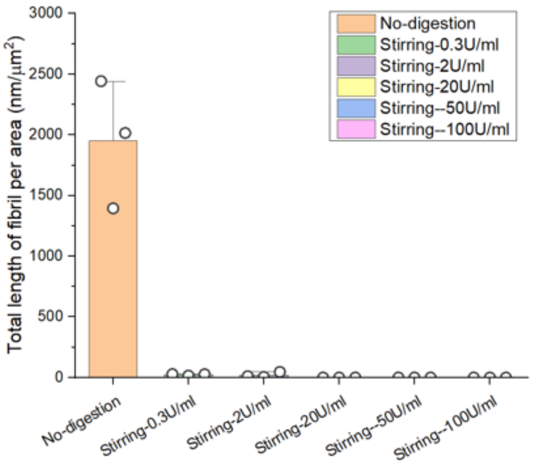

	Total length of fibril per area (nm/µmΛ2)			Average (nm/µmΛ2)
No digestion	2015.20	1395.10	2442.46	1950.92±526.63
Shaking-0.3U/ml	15.90687	28.70346	28.43687	24.35±7.31
Shaking-2U/ml	5.15418	10.66382	46.8535	20.89±22.65
Shaking-20U/ml	0	0	0	0
Shaking-50U/ml	0	0	0	0
Shaking-100U/ml	0	0	0	0

Supplementary Figure 13. AFM statistical analysis of β-lg amyloid fibrils after gastrointestinal digestion in the stirring INFOGEST protocol. N=3 independent experiments. The total length of fibril per area were calculated at different concentrations of trypsin ranging from 0.3 U/mL to 100 U/mL. Data are presented as mean±SD.

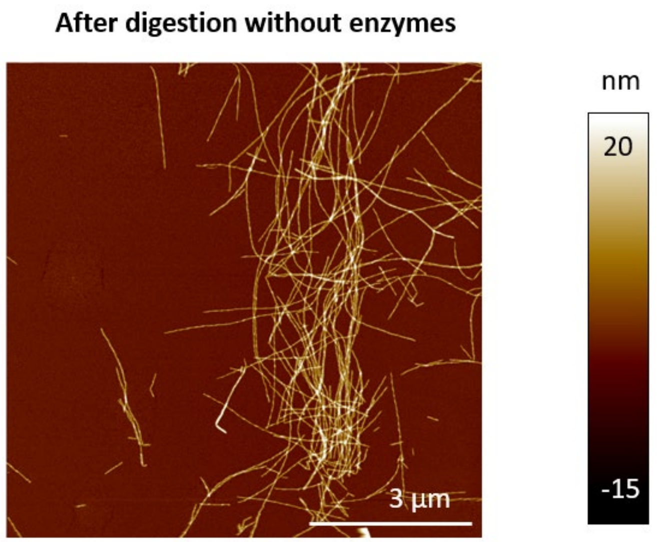

Supplementary Fig. 14. AFM image of β-lg amyloid fibrils after gastrointestinal digestion without digestive enzymes. The fibrils remained intact overall but tended to entangle together which is believed due to the enlarged the ionic strength by mixing with the SGF and SIF buffer.

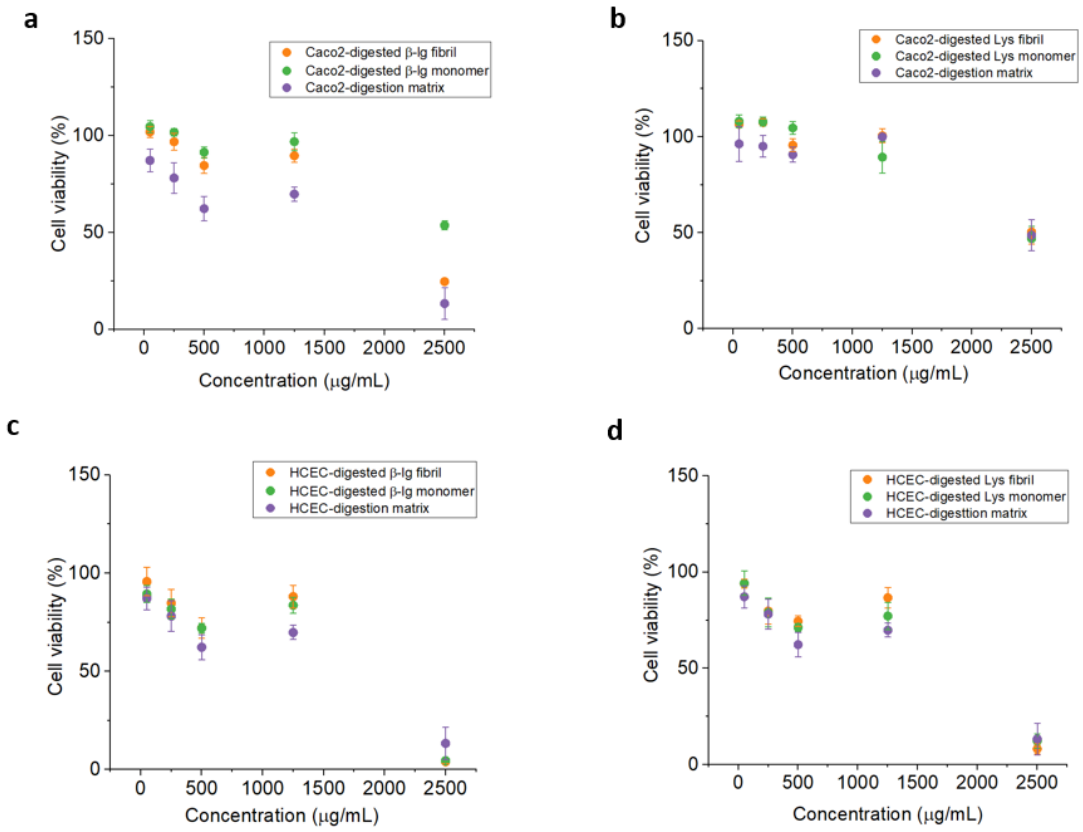

Supplementary Figure 15. Impact of digested fibrils, digested monomer or digestion matrix on viability of human cell lines. (a) BLG in Caco2 cells; (b) Lys in Caco-2 cells; (c) BLG in HCEC cells; (d) Lys in HCEC cells. Cell viability was evaluated on the basis of quantification of ATP present as an indicator of metabolically active cells, performed using the CellTiter-Glo luminescent cell viability assay. Cells were exposed for 4 h. Each cell viability measurement was performed in triplicate and repeated three times. The % cell viability values for exposed cells were normalized to corresponding values for the same cells incubated in the same cell culture medium without addition of the test substances or matrix. Results are shown as mean values+/-SD. N=3 biological replicates.

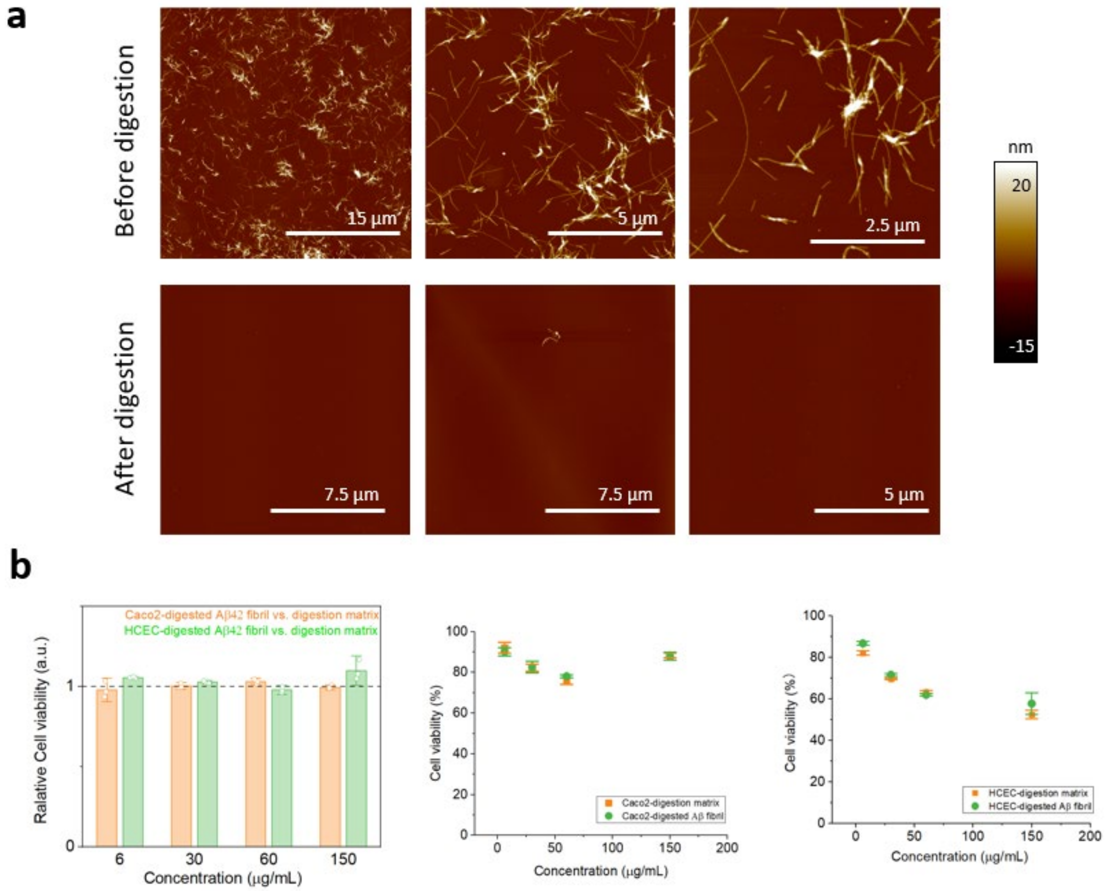

Supplementary Figure 16. The digestion of Aβ42 fibrils and the effect of in-vitro digested protein amyloids on cell viability. (a) The digestion of Aβ42 fibrils upon the trypsin enzyme activity of 0.3U/mL. The Aβ42 fibrils were obtained at the concentration of 50 µM in the buffer of 1:1mixture of 0.1% NH4OH and 100 mM Tris buffer (with 0.02% NaN3 and pH 7.4). (b) the relative Caco2 and HCEC cell viability in the left panel treated with digested fibril vs. digestion buffer at the treatment concentration of 6, 30, 60 and 150 µg/mL. Cell viability was evaluated on the basis of quantification of ATP present as an indicator of metabolically active cells, performed using the CellTiter-Glo luminescent cell viability assay. Cells were exposed for 4 h. Each cell viability measurement was performed in triplicate and repeated three times. The cell viability values for exposed cells were normalized to corresponding values for the same cells incubated in the same cell culture medium without addition of Aβ42 and enzymes. Results are shown as mean values+/-SD.

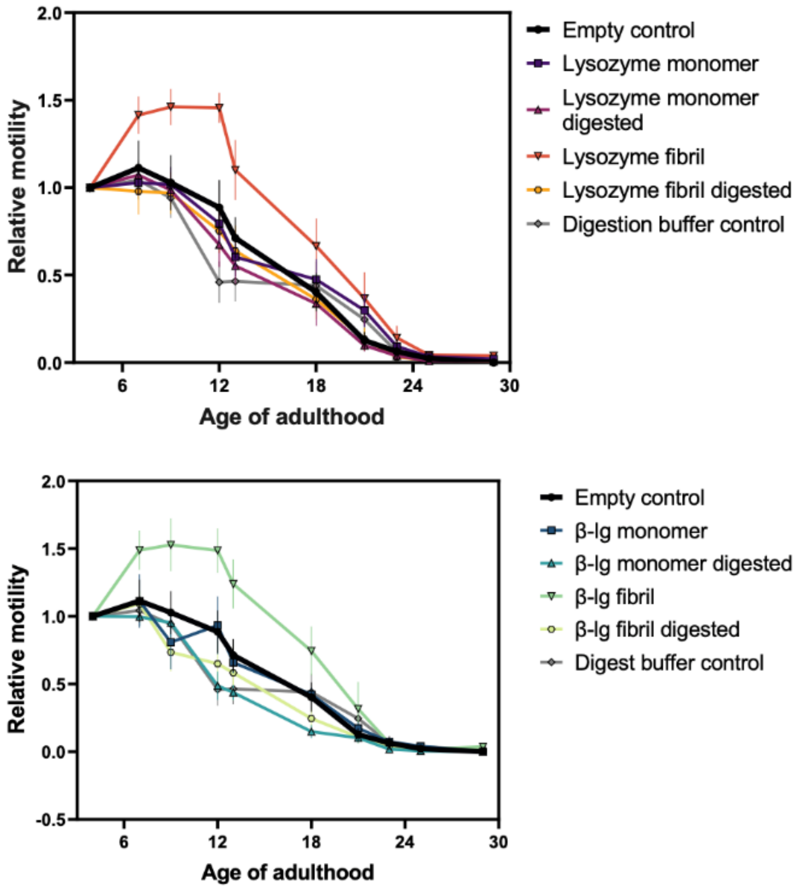

Supplementary Figure 17. Duplicate experiment of *C. elegans* motility, demonstrating higher relative motilities / improved health when *C. elegans* is fed with β-lg and lysozyme fibrils. N=3 biological replicates. Data are presented as mean±SEM.

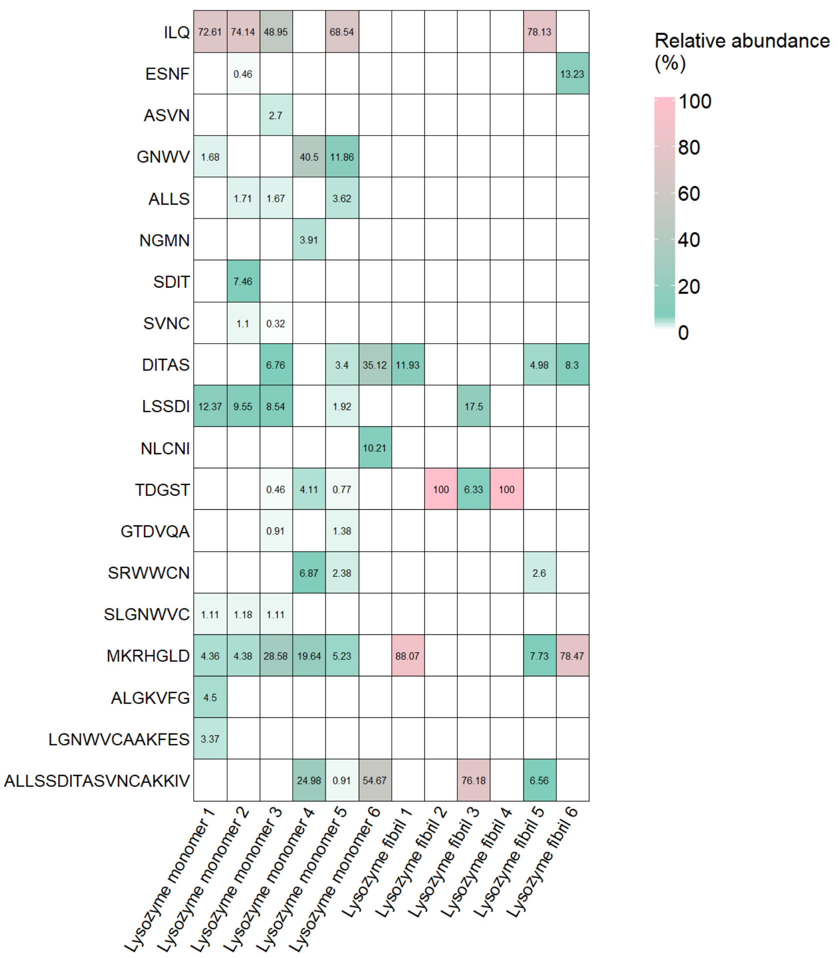

Supplementary Figure 18. Identified digested polypeptides of lysozyme monomer and fibrils in intestine of mice after 4 h oral administration. Color scale represents the intensity of the relative abundance of digested polypeptides.

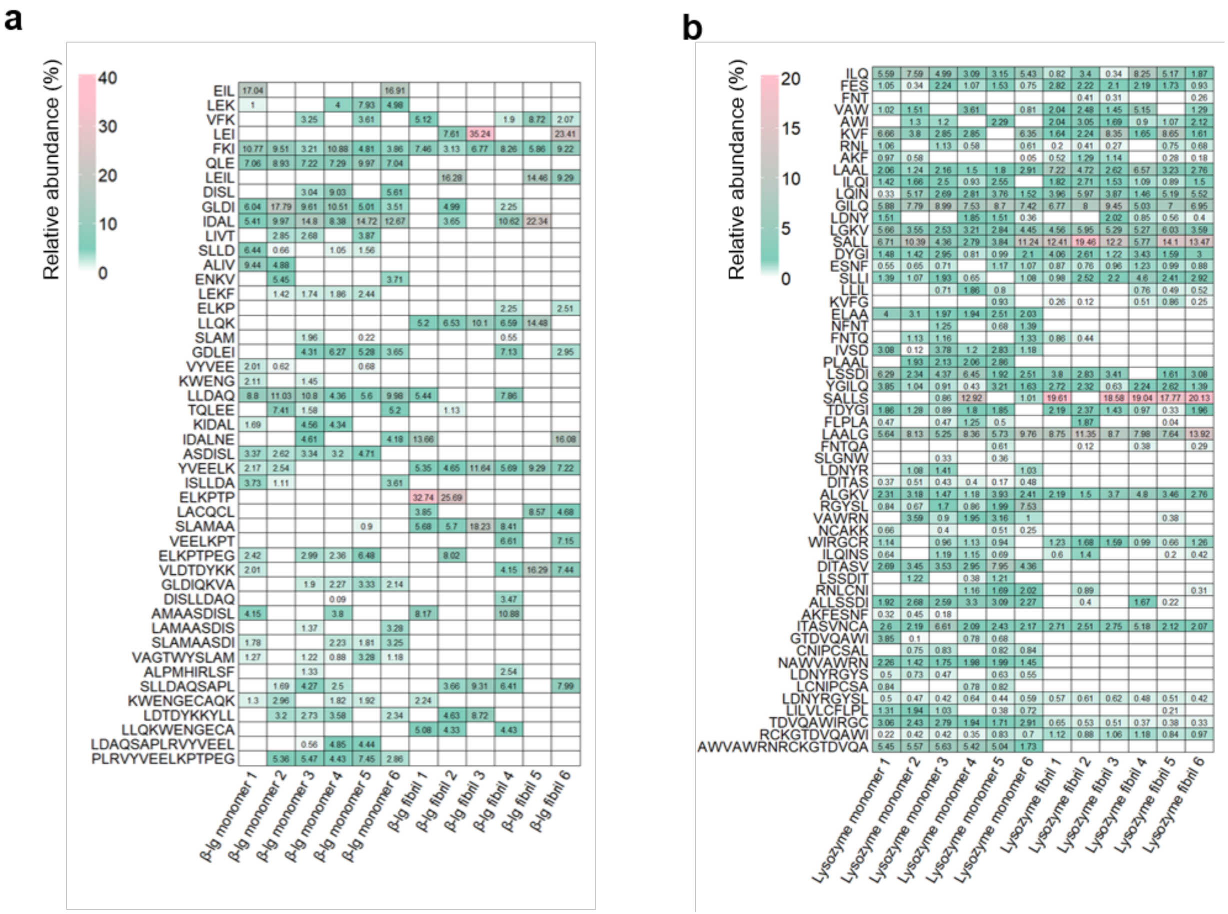

Supplementary Figure 19. Identified digested polypeptides of β-lg (a) and lysozyme (b) monomer and fibrils in colon of mice after 4 h oral administration. Color scale represents the intensity of the relative abundance of digested polypeptides.

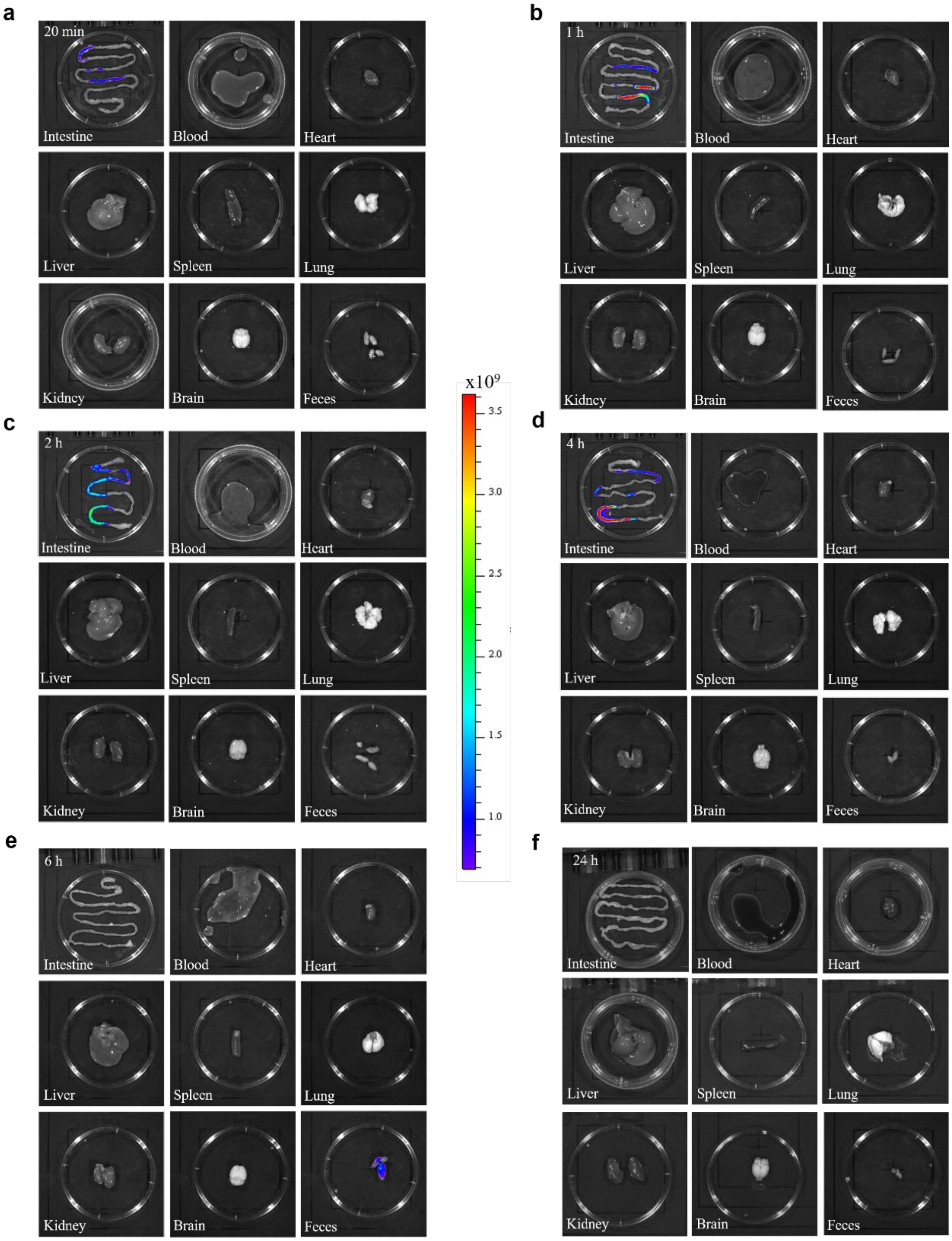

Supplementary Figure 20. Fluorescence images of 5-DTAF-labeled β-lg fibril distribution in main organs and feces of mice after 20 min (a), 1h (b), 2 h (c), 4 h (d), 6 h (e), and 24 h (f) of oral administration.

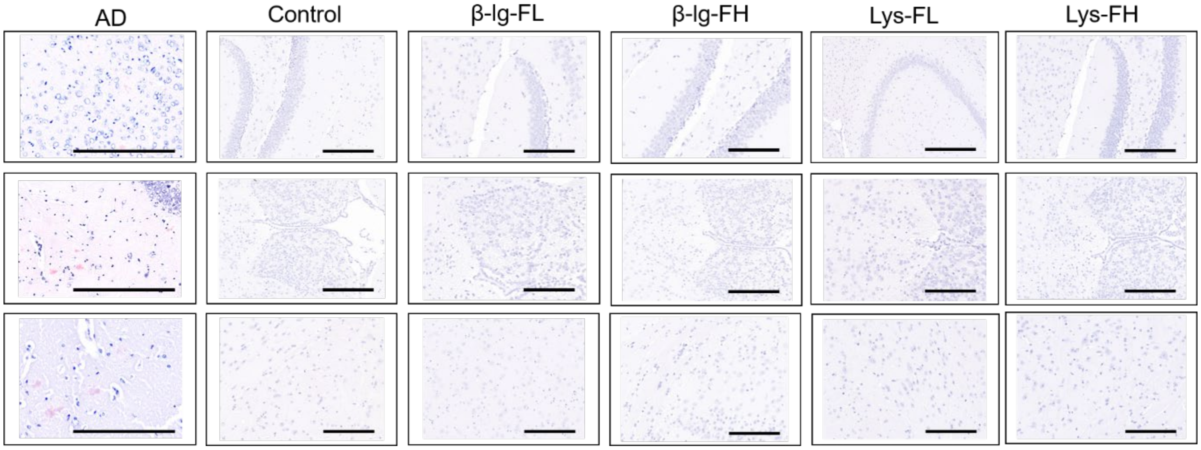

Supplementary Figure 21. Micrographs of CR-stained brain tissue sections from different mice groups after 30 d of feeding food amyloid (where F stands for fibril, H and L stand for high and low dosage). Scale bars represent 200 μm.

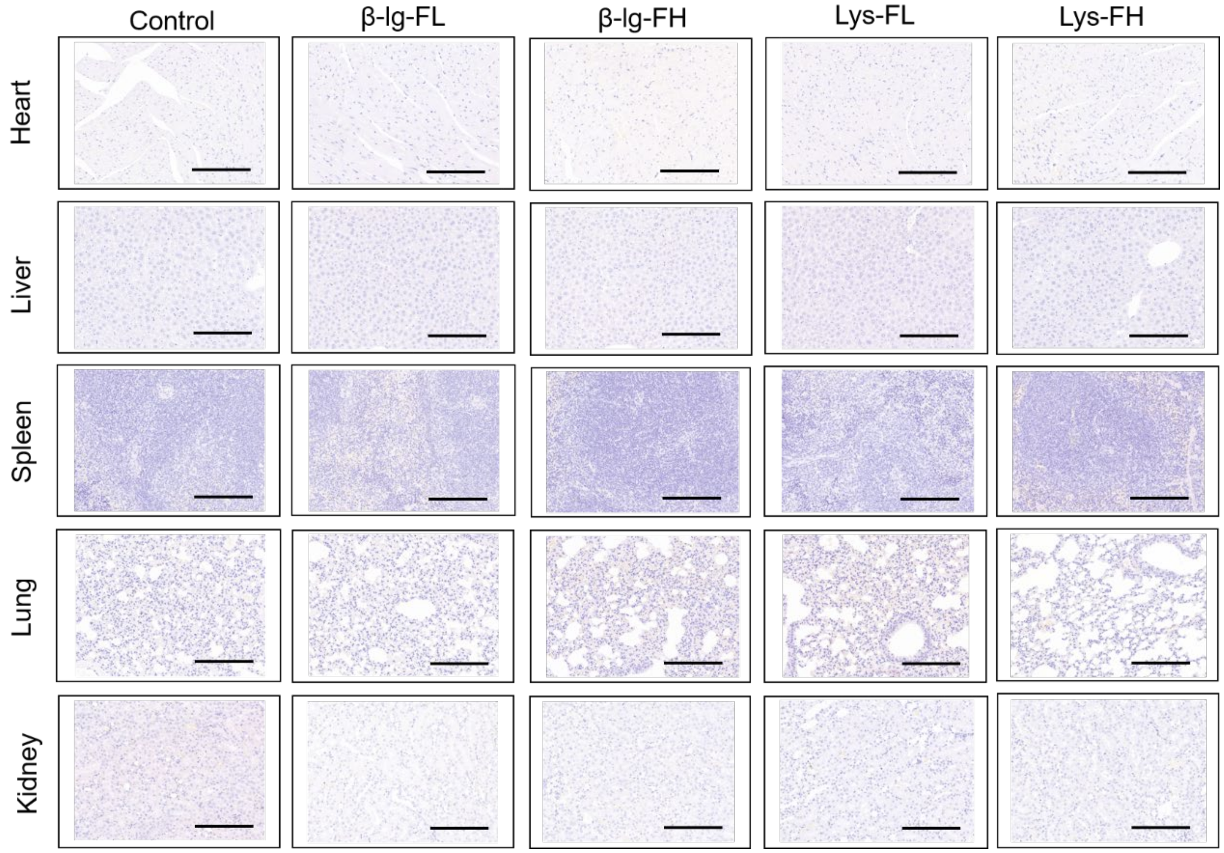

Supplementary Figure 22. Micrographs of CR-stained major organ tissue sections from different mice groups after 30 d of feeding food amyloid (where F stands for fibril, H and L stand for high and low dosage). Scale bars represent 200 μm.

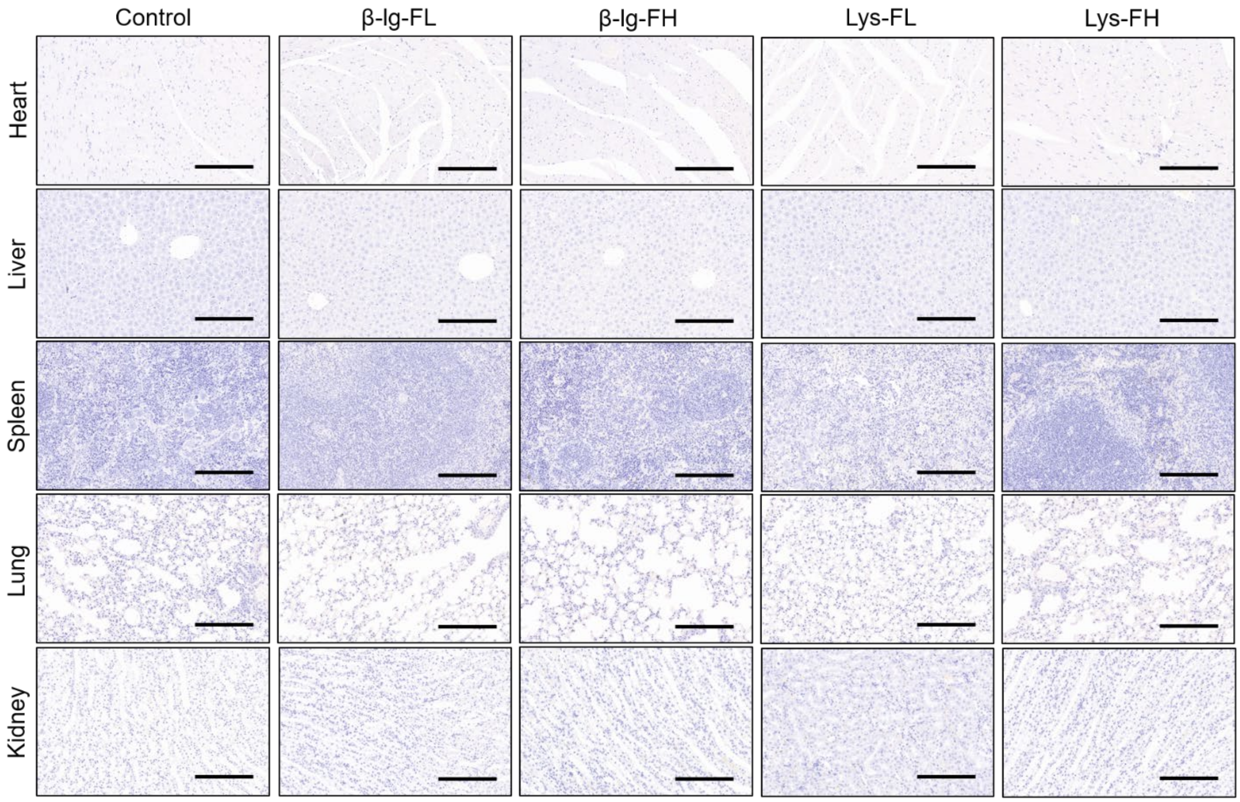

Supplementary Figure 23. Micrographs of CR-stained major organ tissue sections from different mice groups after 60 d of feeding food amyloid (where F stands for fibril, H and L stand for high and low dosage). Scale bars represent 200 μm.

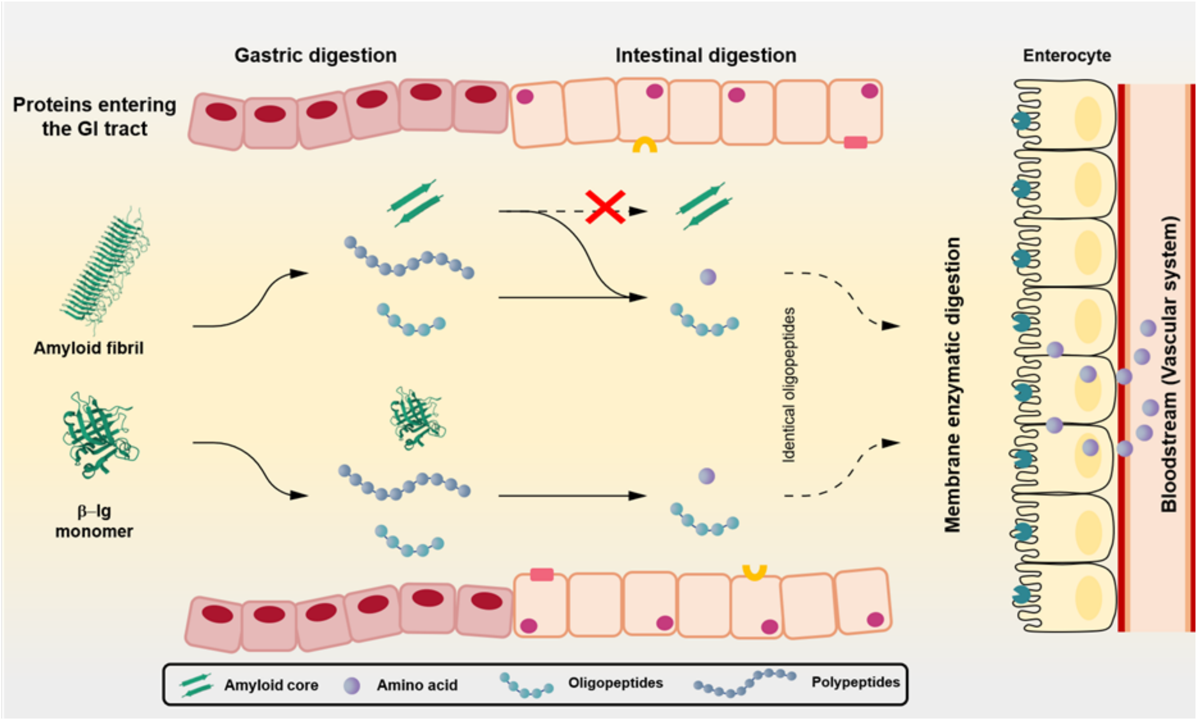

Supplementary Figure 24. Schematic model of β-lg monomer and amyloid fibrils after entering in the gastrointestinal tract.

Supplementary Table 1. Overview of the molecular mass (Da) in the reflector mode of MALDI-MS spectra of the peptides derived from intestinal digested β-lg monomers (upper table) and β-lg amyloid fibril (lower table). The yellow color identifies common peaks in both systems, green color peaks found only in one system, and red peaks those sequences not belonging to β-lg primary structure.

The two green peaks at Peak-1 (815.45 Da) and Peak-16 (1354.71 Da) found in the digested β-lg fibrils (lower table) but not in the digested monomers (upper table) can be sequenced into either VRTPEVD, or NENKVLV or VEELKPT for 815.45 Da and as YSLAMAASDISLL or QKVAGTWYSLAM for 1354.71 Da. We find the VRTPEVD in the Peak-15 from the monomer (**VRTPEVD**D), NENKVLV in the Peak-53 from the monomer (IDAL**NENKVLV**LD), VEELKPT in the Peak-75 from the monomer (RVY**VEELKPT**PEGDLE). Furthermore, we find Y**SLAMAASDISLL** in the Peak-75$_b$ from the monomer (**SLAMAASDISLL**DAQSAPL), and Q**KVAGTWYSLAM** in the Peak-53$_b$ from the monomer (**KVAGTWYSLAM**AAS). The subscript b indicates homologue possible sequences corresponding to the same m/z peak in the reflector mode.

Peaklist (intestinal digested β-lg monomers):

Peak	Mass	Intensity	Peak	Mass	Intensity	Peak	Mass	Intensity	Peak	Mass	Intensity	Peak	Mass	Intensity
1	603.217	529.223	2	609.230	471.709	3	622.063	1126.373	4	645.239	721.532	5	653.120	745.222
6	656.075	982.878	7	672.050	627.234	8	678.370	470.134	9	700.345	1099.990	10	723.345	2112.598
11	739.319	570.177	12	745.328	590.261	13	834.471	789.252	14	865.380	526.304	15	930.483	921.831
16	969.564	616.011	17	983.515	677.672	18	985.519	1912.590	19	993.352	1678.243	20	998.195	1167.329
21	1041.587	2163.722	22	1051.360	1011.431	23	1059.533	4112.574	24	1063.567	1901.394	25	1079.542	685.755
26	1085.552	674.789	27	1098.604	830.306	28	1100.624	822.084	29	1105.642	1080.633	30	1117.533	1444.834
31	1121.457	1564.328	32	1124.493	621.961	33	1130.574	2570.831	34	1139.516	741.471	35	1162.667	765.764
36	1179.464	1847.839	37	1204.656	1551.644	38	1242.452	1546.491	39	1245.634	21450.852	40	1257.681	633.764
41	1267.612	1388.877	42	1276.713	1606.007	43	1313.492	3936.697	44	1320.812	693.595	45	1370.548	1596.224
46	1372.712	8722.160	47	1379.518	1322.250	48	1385.744	756.767	49	1401.743	1056.314	50	1435.740	641.677
51	1441.591	3088.445	52	1450.557	871.357	53	1455.779	12818.493	54	1477.761	1123.584	55	1489.802	803.885
56	1499.598	2153.259	57	1500.813	19767.711	58	1507.735	752.318	59	1508.561	3281.540	60	1554.640	1257.151
61	1568.861	888.096	62	1569.647	831.125	63	1605.803	576.850	64	1618.845	2042.522	65	1636.662	2945.761
66	1682.739	1050.647	67	1735.894	585.658	68	1749.708	804.046	69	1762.916	1161.633	70	1764.718	798.021
71	1789.848	1151.980	72	1805.844	15998.747	73	1821.837	1264.137	74	1840.961	1050.302	75	1873.936	575.671
76	1877.804	636.762	77	1930.962	1293.386	78	2025.824	1263.894	79	2060.004	1721.467	80	2146.057	730.577
81	2264.098	732.681	82	2283.138	739.073	83	2293.167	449.104	84	2309.162	451.180	85	2371.173	1111.265
86	2427.161	510.417	87	2484.192	714.835	88	2502.252	500.385	89	2638.302	500.977	90	2654.298	5950.509
91	2670.289	1085.505	92	2676.277	486.235	93	2711.322	452.118	94	2728.324	437.691	95	2770.349	530.862
96	2825.373	619.551	97	2884.435	449.940	98	2983.506	370.414	99	3013.484	361.751	100	4655.182	209.546

Peaklist (intestinal digested β-lg fibrils):

Peak	Mass	Intensity	Peak	Mass	Intensity	Peak	Mass	Intensity	Peak	Mass	Intensity	Peak	Mass	Intensity
1	815.452	238.102	2	930.489	845.716	3	969.562	668.090	4	1059.540	1232.294	5	1098.613	639.551
6	1105.645	464.812	7	1112.567	1005.423	8	1130.581	990.805	9	1162.667	351.192	10	1204.665	344.243
11	1242.455	493.961	12	1276.717	824.329	13	1313.498	665.976	14	1326.737	425.648	15	1353.494	377.019
16	1354.710	932.857	17	1377.514	392.294	18	1385.747	447.782	19	1435.743	334.857	20	1489.808	416.643
21	1569.659	369.709	22	1627.662	303.885	23	1789.860	382.397	24	1805.860	4232.172	25	1821.859	369.263
26	1840.970	337.542	27	1873.952	291.859	28	1916.167	285.185	29	1930.975	543.228	30	2025.842	410.787
31	2060.025	1006.562	32	2137.101	237.837	33	2146.081	572.876	34	2264.121	327.610	35	2265.311	218.201
36	2283.165	374.299	37	2293.192	290.334	38	2309.187	305.770	39	2371.205	918.921	40	2427.201	235.463
41	2485.253	269.980	42	2502.283	250.464	43	2638.333	202.699	44	2654.331	2630.364	45	2670.322	461.713
46	2685.384	205.118	47	2727.360	193.678	48	2770.377	213.703	49	2800.399	189.984	50	2826.414	259.355
51	2884.473	177.885	52	2910.493	159.999	53	3013.517	156.093	54	3025.528	172.133	55	4655.263	104.737

Supplementary Table 2. Relative motility of *C. elegans* fed with monomer, amyloid fibrils, and *in-vitro* digested amyloid fibrils of β-lg and lysozyme.

Days of adulthood	Control			β-lg monomer	β-lg monomer digested	β-lg fibril	β-lg fibril digested	Lysozyme monomer	Lysozyme monomer digested	Lysozyme fibril	Lysozyme fibril digested	Digest buffer control
3	1	1	1	1	1	1	1	1	1	1	1	1
5	0.995	1.060	1.072	0.982	1.017	1.371	1.094	1.148	1.118	1.078	1.123	1.164
7	0.859	0.972	0.997	0.819	0.977	1.089	0.953	1.029	0.951	1.027	1.047	1.045
10	0.631	0.754	0.795	0.672	0.740	0.838	0.861	0.825	0.767	0.918	0.783	0.693
12	0.579	0.640	0.578	0.481	0.556	0.795	0.590	0.682	0.557	0.887	0.669	0.490
14	0.805	0.821	0.865	0.565	0.689	0.810	0.738	0.708	0.792	0.939	0.688	0.775
16	0.605	0.731	0.763	0.639	0.595	1.074	0.615	0.697	0.611	1.087	0.761	0.684
18	0.399	0.544	0.671	0.535	0.363	1.188	0.508	0.531	0.598	0.944	0.721	0.484
20	0.562	0.459	0.594	0.559	0.418	0.926	0.439	0.433	0.469	0.742	0.712	0.286
21	0.415	0.254	0.344	0.389	0.284	0.930	0.385	0.389	0.370	0.838	0.405	0.272
22	0.247	0.090	0.076	0.284	0.149	0.834	0.363	0.290	0.243	0.590	0.471	0.189
23	0.160	0.038	0.060	0.136	0.201	0.424	0.118	0.128	0.099	0.512	0.286	0.180
24	0.135	0.008	0.020	0.096	0.048	0.408	0.047	0.096	0.031	0.229	0.151	0.063
25	0.062	0.003	0.014	0.089	0.082	0.403	0.157	0.149	0.066	0.266	0.194	0.039

Supplementary Table 3. Number of Q35 aggregates per worm fed with monomer, amyloid fibrils, and *in-vitro* digested amyloid fibrils of β-lg and lysozyme.

Control	β-lg monomer 1.5 mg/ml	β-lg monomer 0.25mg/ml	β-lg fibril 1.5 mg/ml	β-lg fibril 0.25mg/ml	β-lg fibril digested 0.25mg/ml	Lysozyme fibril 0.25mg/ml	Lysozyme fibril 1.5 mg/ml	Lysozyme digested 0.25mg/ml	Lysozyme monomer 0.25mg/ml	Lysozyme monomer 1.5 mg/ml
10	4	5	6	9	6	7	14	10	5	3
5	8	6	7	9	7	2	11	7	8	7
9	12	12	8	10	9	9	6	2	6	17
6	12	4	7	10	6	7	7	10	8	6
5	8	9	9	7	6	2	7	4	2	5
9	11	13	10	2	9	11	16	8	7	8
6	11	9	14	8	11	6	10	10	10	8
10	1	5	9	11	3	9	11	5	13	11
7	3	7	10	9	7	6	10	12	7	12
9	9	7	11	10	4	8	9	15	4	7
3	8	3	6	8	6	12	7	7	9	20
2	9	9	9	10	6	6	7	13	8	9
6	9	15	6	6	12	7	9	6	5	5
8	8	11	9	4	14	3	12	9	8	10
12	10	10	10	8	6	5	7	2	9	3
3	12	6	8	7	7	10	10	6	6	9
7	12	6	9	8	7	10	10	8	10	6
13	2	13	12	11	6	8	12	3	2	8
5	6	7	7	15	8	6	6	6	9	7
7	6	7	7	6	9	14	9	6	5	7
5	11	4	7	8	9	8	13	7	1	7
5	4	5	6	9	14	9	11	6	9	7
5	17	13	8	8	8	11	9	7	7	5
4	5	8	11	18	7	6	10	12	5	5
8	6	7	13	8	6	6	5	6	9	7
7	12	9	9	6	7	8	9	5	9	19
7	13	7	16	5	7	4	9	7	7	9
6	4	4	11	7	9	6	9	8	5	6
9	5	5		6	6	10	11	8	4	7
12	7			6	9	6	15	6	7	5
14				8		7	9	10		
10				13			11			
11				15			15			
8				9						
3										
3										
9										
11										
8										
5										

Supplementary Table 4. Detection of peptides in mice serum after 1h digestion*

Sequence	Length	Mass	Location
MHIRLSFNPTQ	11	1342.6816	161-171
KIDALNE	7	801.42323	99-105

*No peptides benchmarked against sequences of β-lg and lysozyme were detected in mice serum after 1h digestion. The only two identified oligopeptides reported in Table S3 which could match β-lg sequences were found in the blood serum of all mice groups: mice fed with β-lg monomers, mice fed with β-lg amyloids and control mice. All mice were starved for 24 h prior to administration. This demonstrates that the presence of these two oligopeptide sequences is not coming from exogenous β-lg amyloids diet.
