# Supplementary material for: Food amyloid fibrils are safe nutrition ingredients based on in-vitro and in-vivo assessment
Source: Nat Commun. 2023 Oct 26;14:6806. doi: 10.1038/s41467-023-42486-x (PMC10603083; doi:10.1038/s41467-023-42486-x)
Supplement: Supplementary file 3 — Reporting Summary [file 41467_2023_42486_MOESM3_ESM.pdf]

nature portfolio

Corresponding author(s): Raffaele Mezzenga, Jiangtao Zhou

Last updated by author(s): Sep 18, 2023

Reporting Summary

Nature Portfolio wishes to improve the reproducibility of the work that we publish. This form provides structure for consistency and transparency in reporting. For further information on Nature Portfolio policies, see our Editorial Policies and the Editorial Policy Checklist.

Statistics

For all statistical analyses, confirm that the following items are present in the figure legend, table legend, main text, or Methods section.

n/a	Confirmed	
☐	☒	The exact sample size (*n*) for each experimental group/condition, given as a discrete number and unit of measurement
☐	☒	A statement on whether measurements were taken from distinct samples or whether the same sample was measured repeatedly
☐	☒	The statistical test(s) used AND whether they are one- or two-sided<br>*Only common tests should be described solely by name; describe more complex techniques in the Methods section.*
☐	☒	A description of all covariates tested
☐	☒	A description of any assumptions or corrections, such as tests of normality and adjustment for multiple comparisons
☐	☒	A full description of the statistical parameters including central tendency (e.g. means) or other basic estimates (e.g. regression coefficient) AND variation (e.g. standard deviation) or associated estimates of uncertainty (e.g. confidence intervals)
☐	☒	For null hypothesis testing, the test statistic (e.g. *F*, *t*, *r*) with confidence intervals, effect sizes, degrees of freedom and *P* value noted<br>*Give P values as exact values whenever suitable.*
☒	☐	For Bayesian analysis, information on the choice of priors and Markov chain Monte Carlo settings
☒	☐	For hierarchical and complex designs, identification of the appropriate level for tests and full reporting of outcomes
☒	☐	Estimates of effect sizes (e.g. Cohen's *d*, Pearson's *r*), indicating how they were calculated

Our web collection on statistics for biologists contains articles on many of the points above.

Software and code

Policy information about availability of computer code

Data collection	Bruker NanoSurfaces, Fiberapp, Jasco spectra manager, Infinite M200 pro, Zeiss AxioImager Z2, NIS Elements Nikon software
Data analysis	Excel, Origin Pro, Nanoscope Analysis 1.5, ImageJ, FlexControl

For manuscripts utilizing custom algorithms or software that are central to the research but not yet described in published literature, software must be made available to editors and reviewers. We strongly encourage code deposition in a community repository (e.g. GitHub). See the Nature Portfolio guidelines for submitting code & software for further information.

Data

Policy information about availability of data

All manuscripts must include a data availability statement. This statement should provide the following information, where applicable:

- Accession codes, unique identifiers, or web links for publicly available datasets
- A description of any restrictions on data availability
- For clinical datasets or third party data, please ensure that the statement adheres to our policy

The data used to reproduce the results and to support the findings are available within the article and Supplementary Information file. Unprocessed data and blots are provided in the Source Data file, and Source Data are provided with this paper. Extra data are available from the corresponding authors upon request

Human research participants

Policy information about studies involving human research participants and Sex and Gender in Research.

Reporting on sex and gender	N/A
Population characteristics	N/A
Recruitment	N/A
Ethics oversight	N/A

Note that full information on the approval of the study protocol must also be provided in the manuscript.

Field-specific reporting

Please select the one below that is the best fit for your research. If you are not sure, read the appropriate sections before making your selection.

☒ Life sciences ☐ Behavioural & social sciences ☐ Ecological, evolutionary & environmental sciences

For a reference copy of the document with all sections, see nature.com/documents/nr-reporting-summary-flat.pdf

Life sciences study design

All studies must disclose on these points even when the disclosure is negative.

Sample size	All experiments were performed with beta-lactoglobulin amyloid fibrils purified with the same method stated in the manuscript. Final stock amyloid fibrils were of the concentration 0.4 wt.% in the sample size of 10-50 mL.
Data exclusions	No data was excluded.
Replication	Experiments were repeated at least three times, and all attempts were successful.
Randomization	Different batches of amyloid fibrils purified with the same method were randomly used to verify the results of each experiment.
Blinding	Blinding was not relevant as the investigators followed specific protocols to perform the experiments.

Reporting for specific materials, systems and methods

We require information from authors about some types of materials, experimental systems and methods used in many studies. Here, indicate whether each material, system or method listed is relevant to your study. If you are not sure if a list item applies to your research, read the appropriate section before selecting a response.

Materials & experimental systems

n/a	Involved in the study	
☒	☐	Antibodies
☐	☒	Eukaryotic cell lines
☒	☐	Palaeontology and archaeology
☐	☒	Animals and other organisms
☒	☐	Clinical data
☒	☐	Dual use research of concern

Methods

n/a	Involved in the study	
☒	☐	ChIP-seq
☒	☐	Flow cytometry
☒	☐	MRI-based neuroimaging

Eukaryotic cell lines

Policy information about cell lines and Sex and Gender in Research

Cell line source(s)	Caco2 cells were obtained from the Food Biotechnology group at ETH Zurich. Caco2 cells are epithelial cells isolated from colon tissue derived from a patient with colorectal cadenocarcinoma. They are cultured in DMEM (cat. Nr: 31966 021, ThermoFisher Scientifc, Massachusetts, USA) with 10% FBS (cat. Nr: 10270106, ThermoFisher Scientifc, Massachusetts, USA) 100 U/ml penicillin and 100 µg/ml streptomycin (cat. r.: 15,140,122, ThermoFisher Scientifc, Massachusetts, USA). HCEC-lCT cells were obtained from Jerry Shay from the UT Suthwestern in August 2011. HCEC-lCT cells are a colon epitelial cell line.

	They were cultured in ready mixed full medium (cat. Nr: MHT-039, Evercyte, Vienna, Austria). Caco2 cells were seeded at a density of 5000/cells/well in 100 l medium in 96-well plates (cat Nr: 400096, Bioswisstec, Schhaussen, Switzerland), HCEC-lCT cells were seeded at a density of 2500 cells/well in 100 l medium in 96-well plates (cat. Nr: 734-0079, Corning, New York, USA). ATP was quantified, as an indicator of metabolically active cells (cat. Nr:G7571, Promega, Dubendorf, Schweiz).
Authentication	None of the cell line was authenticated (see above)
Mycoplasma contamination	The cell lines were all tested negative for Mycoplasma contamination
Commonly misidentified lines (See ICLAC register)	No commonly misidentified cell lines were used in the study

Animals and other research organisms

Policy information about studies involving animals; ARRIVE guidelines recommended for reporting animal research, and Sex and Gender in Research

Laboratory animals	Kunming male mice (5 weeks old) , and Mice were housed in enclosures on sawdust bedding at 25 °C and 40% relative humidity including a normal light cycle (12 h dark /12 h light cycle). C. elegans (Strains: TJ1060 spe-9(hc88) I) are studied at day 1 of adulthood
Wild animals	The study did not involve wild animals.
Reporting on sex	Sex was not considered in this study, and only male mice were used.
Field-collected samples	The study did not involve samples collected from fields.
Ethics oversight	The Laboratory Animal Committee (LAC) of South China University ofTechnology provided the guidance on the study protocol

Note that full information on the approval of the study protocol must also be provided in the manuscript.
