# Supplementary material for: Food amyloid fibrils are safe nutrition ingredients based on in-vitro and in-vivo assessment
Source: Nat Commun. 2023 Oct 26;14:6806. doi: 10.1038/s41467-023-42486-x (PMC10603083; doi:10.1038/s41467-023-42486-x)
Supplement: Supplementary file 4 — Source Data [file 41467_2023_42486_MOESM4_ESM.zip › Source Data/Upcropped data_Figure 1b-c.pptx]

## Slide 1
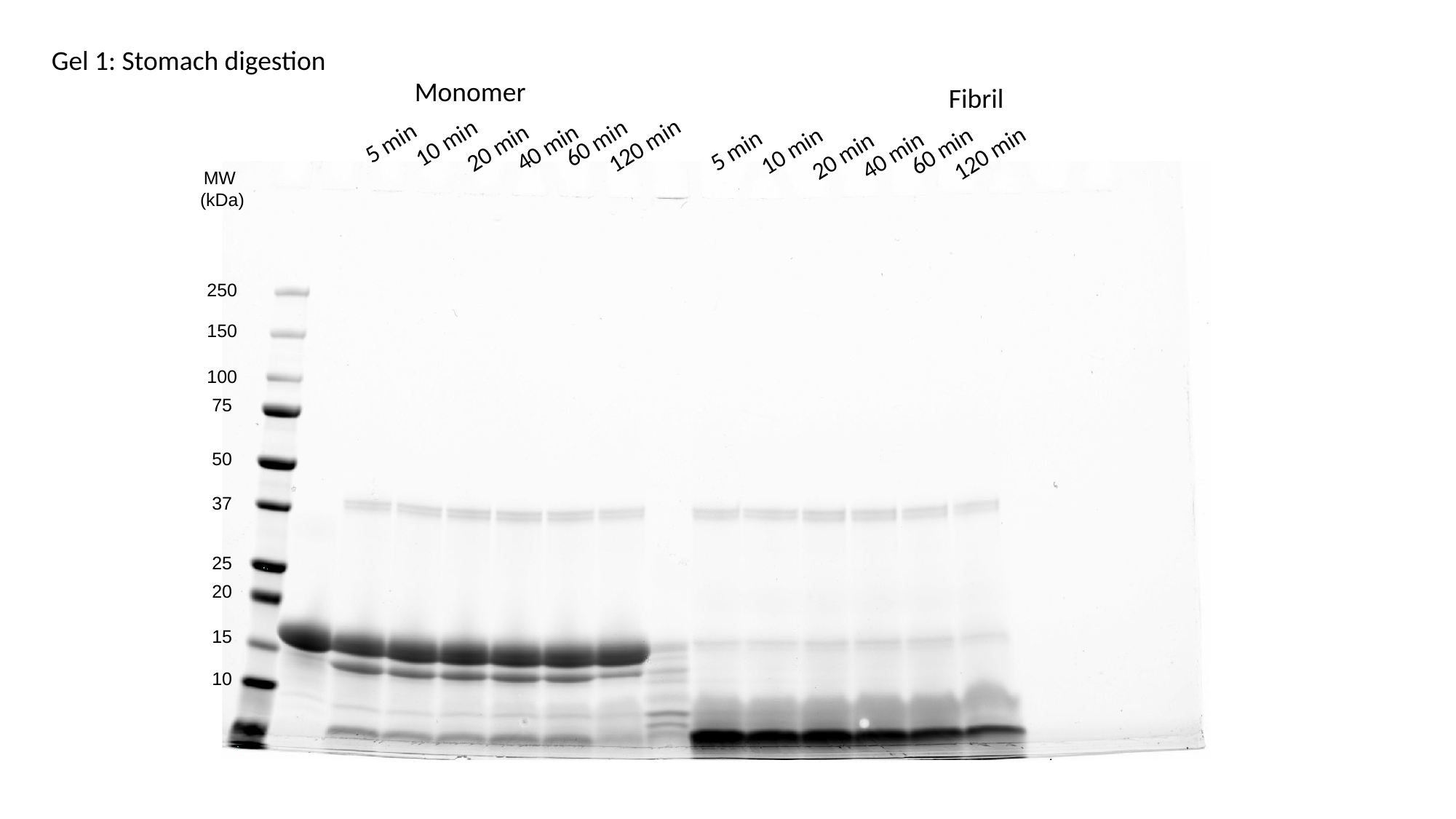

Gel 1: Stomach digestion
Monomer
Fibril
5 min
60 min
10 min
120 min
40 min
20 min
5 min
60 min
10 min
120 min
40 min
20 min
MW
(kDa)
250
150
100
75
50
37
25
20
15
10

## Slide 2
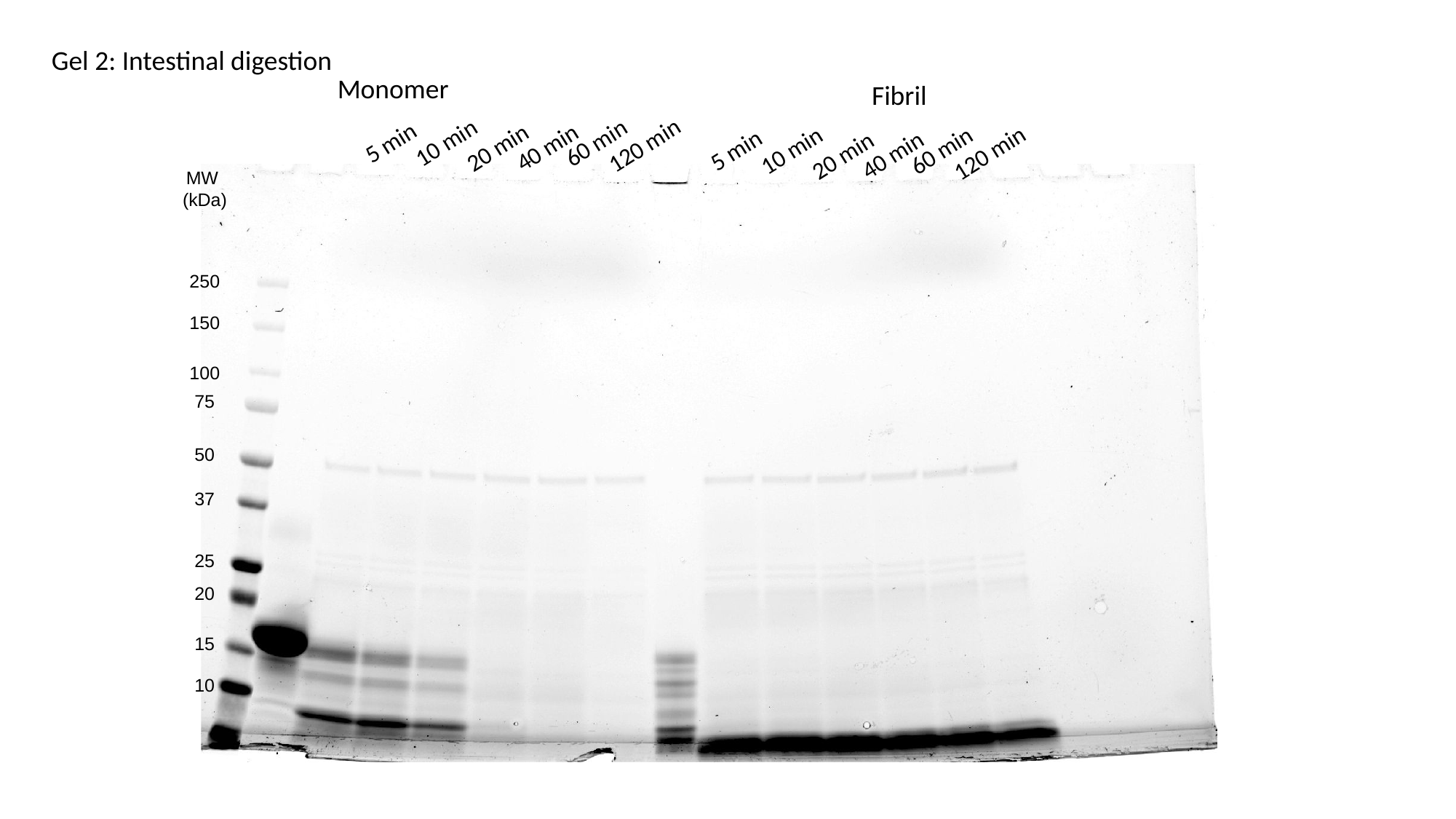

Gel 2: Intestinal digestion
Monomer
Fibril
5 min
60 min
10 min
120 min
40 min
20 min
5 min
60 min
10 min
120 min
40 min
20 min
MW
(kDa)
250
150
100
75
50
37
25
20
15
10
